# Genomic alterations underlie a pan-cancer metabolic shift associated with tumour hypoxia

**DOI:** 10.1186/s13059-016-0999-8

**Published:** 2016-06-29

**Authors:** Syed Haider, Alan McIntyre, Ruud G. P. M. van Stiphout, Laura M. Winchester, Simon Wigfield, Adrian L. Harris, Francesca M. Buffa

**Affiliations:** Computational Biology and Integrative Genomics, Department of Oncology, University of Oxford, Oxford, UK; Molecular Oncology Laboratories, Department of Oncology, The Weatherall Institute of Molecular Medicine, University of Oxford, Oxford, UK

**Keywords:** Cancer, Integrative biology, Metabolism, Tumour microenvironment

## Abstract

**Background:**

Altered metabolism is a hallmark of cancer. However, the role of genomic changes in metabolic genes driving the tumour metabolic shift remains to be elucidated. Here, we have investigated the genomic and transcriptomic changes underlying this shift across ten different cancer types.

**Results:**

A systematic pan-cancer analysis of 6538 tumour/normal samples covering ten major cancer types identified a core metabolic signature of 44 genes that exhibit high frequency somatic copy number gains/amplifications (>20 % cases) associated with increased mRNA expression (*ρ >* 0.3, *q* < 10^−3^). Prognostic classifiers using these genes were confirmed in independent datasets for breast and kidney cancers. Interestingly, this signature is strongly associated with hypoxia, with nine out of ten cancer types showing increased expression and five out of ten cancer types showing increased gain/amplification of these genes in hypoxic tumours (*P* ≤ 0.01). Further validation in breast and colorectal cancer cell lines highlighted squalene epoxidase, an oxygen-requiring enzyme in cholesterol biosynthesis, as a driver of dysregulated metabolism and a key player in maintaining cell survival under hypoxia.

**Conclusions:**

This study reveals somatic genomic alterations underlying a pan-cancer metabolic shift and suggests genomic adaptation of these genes as a survival mechanism in hypoxic tumours.

**Electronic supplementary material:**

The online version of this article (doi:10.1186/s13059-016-0999-8) contains supplementary material, which is available to authorized users.

## Background

The role of the microenvironment in driving tumour progression is increasingly recognised. Hypoxia is one of the key physiological and microenvironmental differences between tumour and normal tissues; it induces DNA amplification and damage, whilst reducing repair [1]. Moreover, hypoxia along with acidosis increases clonal selection, resulting in aggressive cancer phenotypes. A crucial mechanism underlying such evolution in a highly dynamic tumour microenvironment is metabolic reprogramming of tumour cells [2–5]. Metabolic adaptation is an emerging hallmark of cancer [6]. It has also been recognised that altered gene regulatory networks in cancer drive the activity of metabolic pathways (reviewed in [4]). Although there are some well-described mutations, e.g. of *IDH1* and *IDH2* [7–10], this is not the case for the vast majority of such genes. Thus, the complex landscape of oncogenic activity of metabolic pathways is far from understood. Therefore, there is an urgent need for understanding the genetic basis of regulation of metabolic genes, characterising their role in driving tumour growth and assessing their potential as new therapeutic targets.

Recent studies have examined transcriptional deregulation in metabolic pathways across different cancers [11–13], identifying candidate drivers of cancer metabolic adaptation. Although these studies highlight the molecular underpinnings of altered metabolism, the mechanisms driving the metabolic shift and clinical potential of metabolic drivers remain obscure. For instance, changes in mRNA abundance can arise from either altered transcriptional machinery or genomic aberrations. Therefore, in order to elucidate mechanistic insights into the dysregulated metabolism of cancer cells and identify potential drivers of this hallmark, it is crucial to study genomic aberrations alongside transcriptional changes. Here, we hypothesise that metabolic requirements in the hypoxic tumour microenvironment are driving selection of genomic alterations leading to genetic heterogeneity as well as transcriptional deregulation. To test this hypothesis high-resolution genomic and transcriptomic profiles of metabolic transporters and enzymes in tumour and normal tissue samples are necessary, as well as associated clinical annotations. Courtesy of projects such as The Cancer Genome Atlas (TCGA), the International Cancer Genome Consortium [14] (ICGC) and Metabric [15], genomic, transcriptomic, epigenomic and proteomic profiles across multiple tumour types have been generated in thousands of well-annotated human cancer samples. Thus, using these profiles, we conducted a pan-cancer integrated analysis of 6538 matched genomic and transcriptomic profiles covering ten tumour types in a comprehensive collection of 2752 metabolic enzyme and transporter genes. Specifically, we asked whether (1) somatically acquired mutations are independent drivers of metabolic shift, (2) over-expression of metabolic genes [13, 16] is induced by somatically acquired copy-number alterations, (3) metabolic genes are associated with previously reported cancer drivers [17–19] and (4) metabolic genes are associated with tumour hypoxia [20] and (5) are prognostic. We then evaluated the top-ranked key rate-limiting enzyme *SQLE* in a panel of aggressive breast cancers and colorectal cancer cell lines.

## Results

### Pan-cancer metabolic landscape and increased dysregulation in hypoxic tumours

A previously curated list of 2752 metabolic enzyme and transporter genes extracted from the Kyoto Encyclopedia of Genes and Genomes (KEGG) database (Additional file 1 and Additional file 2: Table S1) was considered, offering coverage of over-expressed metabolic genes in cancer tissues and association with stemness as well as aggressive breast cancers [13]. To systematically identify pan-cancer metabolic gene signatures characterising multi-tissue cancer profiles, genomic (DNA somatic mutations and copy number aberrations) and transcriptomic (mRNA abundance) data from 6538 samples spanning ten cancer types [15, 21–29] were analysed (Additional file 1 and Additional file 2: Table S2).

We first asked whether genomic alterations were globally enriched in metabolic genes. We found no difference in the mean frequency of somatic copy number alterations (SCNAs) across all cancers between the 2752 metabolic genes and other genes (*P* = 0.85, two-sided proportion test) and a slight but not significant decrease in the mean frequency of somatic mutations (*P* = 0.06, two-sided proportion test).

Given hypoxia is the main microenvironmental factor associated with dysregulated metabolism [2–5] and genomic instability [1, 30], we asked whether genomic alterations in metabolic genes are associated with tumour hypoxia. To achieve this, we applied a previously validated hypoxia signature [20, 31], which we confirmed to be higher in tumour samples compared with normal tissues for the cancer types considered here (Additional file 1: Figure S1a). We observed an increase in genomic alterations in metabolic genes in hypoxic tumours, with a varying degree of significance depending on the type of alteration and cancer type. Specifically, tumour hypoxia was correlated with the extent of SCNAs in metabolic genes in up to seven of the ten cancer types (*P* < 0.05, one-sided Wilcoxon test; Additional file 1: Figure S1b, c) and with the extent of somatic mutations in three of the ten cancer types (Additional file 1: Figure S1d). This agrees with recent reports of hypoxia-driven transient SCNAs in cancer [32]. We tested this further by looking at the acquisition of early genomic aberrations in a stable diploid human cancer cell line model in vivo, complementing the results obtained from the cancer samples and from models with a higher background of genetic instability. After treatment with the antiangiogenic drug bevacizumab, inducing hypoxia (Additional file 1: Figure S2a), we observed SCNA events exclusive to the treatment group (Additional file 1: Figure S2b, c). Considering the chromosomal stability of this cell line, this represents a significant genetic change. Interestingly, the main alteration was in the copy number of chromosome 12, which is significantly enriched for metabolism genes compared with other chromosomes (*P*_*overlap*_ = 0.03, hypergeometric test; Additional file 1: Figure S2d). Given the variety of cancer types and experimental systems considered, the above results taken together suggest that genomic alterations in metabolic pathways, and particularly in SCNAs, act as a conserved selective pressure in hypoxic tumours.

We then asked if we could identify specific SCNA events linked with over-expression across cancer types; if identified, such events would constitute *candidate metabolism drivers*. This is based on the commonly accepted hypothesis that a strong correlation between DNA and mRNA levels is indicative of a gain of function. Specifically, we selected the top 10 % over-expressed genes with respect to normal tissue, across all tumour types (log_2_ fold change *>*0.5; Additional file 1 and Additional file 2: Table S3), and further limited this group to those which were recurrently gained or amplified (putative) in at least 20 % of samples in each cohort (Additional file 1: Figure S3). Next, we performed a pan-cancer correlation analysis which revealed a total of 277 metabolic genes exhibiting positive correlation across different cancer types (Spearman’s *ρ* > 0.3, *q* < 10^−3^; Additional file 1 and Additional file 2: Tables S4 and S5).

**Fig. 1:**
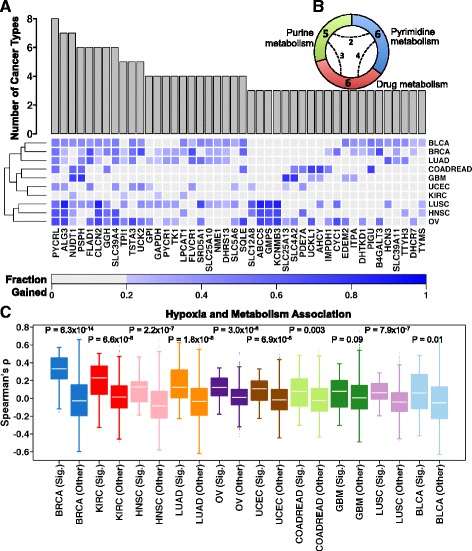
Candidate core pan-cancer drivers of metabolic dysregulation and hypoxia association. **a** For each candidate driver, the cumulative *bar chart* shows the inclusion frequencies representing the number of cancer types whereby mRNA and copy number profiles were correlated. The inclusion criterion was arbitrarily set to a minimum of three cancers. The *heat map* shows the extent of genomic aberrations for the selected candidate drivers as well as the corresponding cancer types. The *intensity* indicates the fraction of the cohort in which a given gene has either a gain or amplification. The *rows* (tumour types) were ordered using hierarchical clustering. **b** The distribution of core candidate metabolic drivers in statistically enriched compartments of metabolic pathways. The *number* in each *segment* indicates the metabolic genes present in that compartment. The *dashed arcs* (with *numbers*) indicate the number of genes shared between the compartments (Additional file 1 and Additional file 2: Table S7). **c** Cancer-wise mRNA correlations (Spearman’s correlation coefficients) of core metabolic signature (44 genes, *Sig.* group) and hypoxia score and compared with the correlations between non-selected metabolic genes (*Other*) and hypoxia score. For each cancer type, the distribution of both sets of correlations was compared using a one-sided Wilcoxon test and the *P* values are displayed. Box plots are sorted (high on the *left* to low on the *right*) by the median correlation coefficient in the signature (*Sig.*) set of scores. To avoid bias, three genes common to the metabolism signature and hypoxia signature and another up to 17 genes (depending upon the cancer type) common to the non-selected metabolic group and hypoxia signature were removed. *BLCA* bladder urothelial carcinoma, *BRCA* breast invasive carcinoma, *LUAD* lung adenocarcinoma, *COADREAD* colorectal adenocarcinoma, *GBM* glioblastoma multiforme, *UCEC* uterine corpus endometrial carcinoma, *KIRC* Kidney renal clear cell carcinoma, *LUSC* lung squamous cell carcinoma, *HNSC* head and neck squamous cell carcinoma, *OV* Ovarian serous cystadenocarcinoma

Interestingly, the frequency of these candidate metabolic drivers varied remarkably between cancers, from six genes (kidney renal cell carcinoma (KIRC)) to 143 genes (ovarian cancer (OV)) (Additional file 1: Figure S4a). The number of candidate metabolic drivers was significantly higher than expected by chance in breast carcinoma (BRCA), colorectal adenocarcinoma (COADREAD), glioblastoma multiforme (GBM) and OV (*P* < 10^−4^; using 10,000 randomly chosen non-metabolic gene sets; see the “Permutation analysis” section in the “Methods”). To identify metabolic processes essential to multiple tumours, we prioritised 44 *core metabolic drivers* with significantly correlated DNA and mRNA changes in cancer samples (Spearman’s *ρ* > 0.3, *q* < 10^−3^) in at least three of the ten tumour types (Fig. 1a; Additional file 1: Figure S4b, c; Additional file 1; Additional file 2: Table S6). The top candidate metabolic drivers were *PYCRL*, *ALG3* and *NUDT1*, which were correlated in at least seven of the ten tumour types. The list also contained *TYMS*, a well-known chemotherapeutic target [33], and another five genes (*GAPDH*, *PYCR1*, *TPI1*, *TSTA3* and *TTYH3*) for which inhibition of expression has been previously shown to reduce cell viability in in vitro and in vivo functional genomics screens [13]. These 44 genes were significantly enriched in five overlapping metabolic processes (Fig. 1b; Additional file 1; Additional file 2: Table S7), including pyrimidine metabolism (six genes, *q* = 1.97 × 10^−4^), drug metabolism (six genes, *q* = 1.97 × 10^−4^) and purine metabolism (five genes, *q* = 0.039).

We then asked if there was an increased association of these 44 genes with hypoxia relative to other metabolic genes. Remarkably, nine of the ten cancer types showed significantly greater positive correlation between the expression of the 44 core metabolic drivers and the hypoxia signature compared with the remainder of the metabolic genes (Fig. 1c; *P* ≤ 0.01, one-sided Wilcoxon test). A similar pattern was maintained in five of the ten cancer types when considering SCNA profiles (Additional file 1: Figure S5a). The association between hypoxia and these 44 genes was also reflected in a significantly elevated fraction of copy number gains for these genes in patients with higher hypoxia in four of the ten cancer types (Additional file 1: Figure S5b; *P* < 0.05, one-sided Wilcoxon test). Conversely, hypoxia was not associated with the extent of somatic mutations in these 44 genes (Additional file 1: Figures S5c). This reflects the lower frequency of somatic mutations in these genes, with an overall mutation rate ranging from 0.056 % (*PYCRL*) to 2.212 % for (*ABCC5*) (Additional file 1 and Additional file 2: Table S8a). Evaluation of all metabolic genes revealed only 12 out of 2752 genes somatically mutated at a mean frequency of >5 % (Additional file 1 and Additional file 2: Table S8b). These results support the hypothesis that a core group of metabolic genes are preferentially selected through genomic gains/amplifications, with rare exceptions such as *IDH1*/*2* [7–10]*.* Equivalent analysis focussing on down-regulated genes with genomic losses resulted in only three recurrent candidates of metabolism (Additional file 1 and Additional file 2: Table S9).

In summary, we identified a core set of 44 metabolic genes which are over-expressed in tumour samples through genomic gains/amplification, exhibit low rates of somatic mutations and demonstrate correlation with tumour hypoxia.

### Association of candidate metabolic drivers with previously reported cancer drivers

**Fig. 2:**
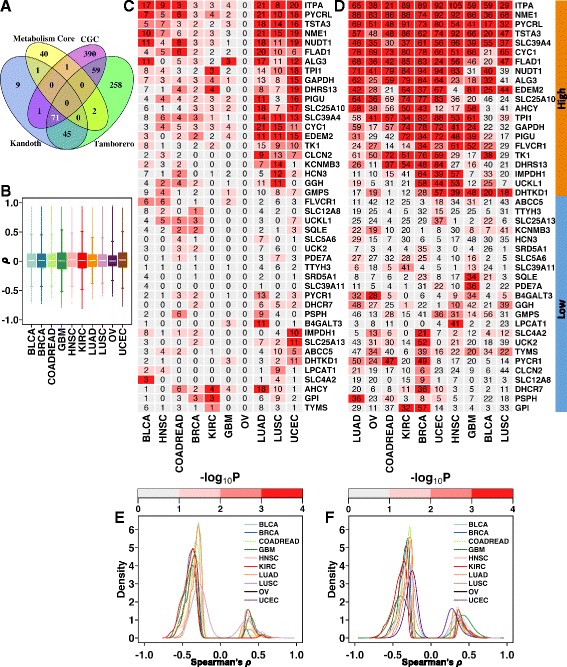
Metabolic compartmental enrichment and candidate drivers of tumourigenesis. **a** Overlap of genes between the core candidate metabolic drivers (*yellow*) and three lists (*blue*, *purple* and *green*) of previously reported cancer drivers [17–19]. The *number in white* (*71*) indicates overlap between the previously reported cancer drivers. **b** Pan-cancer null distributions of Spearman’s correlation coefficients between mRNA profiles of one million random (matched across tumour types) gene pairs. **c**, **d** Pan-cancer enrichment analysis of previously reported cancer-specific drivers (**c**) and the *global* (combined) list of candidate cancer drivers (**d**). The *numbers in the cells* indicate the total number of drivers correlated or anti-correlated with the corresponding metabolic gene (*rows*). The cutoffs for correlation were derived from the null distribution (95th and 5th percentiles) as shown in Fig. 2b. The *cell colour* indicates the probability values of expecting the observed correlation by random chance alone, with high intensities suggesting higher enrichment of previously reported cancer drivers in genes correlated with the candidate metabolic drivers. Tumour types (*columns*) were grouped by hierarchical clustering. The gene-wise (*rows*) clustering groups for the merged list of cancer drivers (**d**) are highlighted in *orange* (high correlation group) and *blue* (low correlation group). **e**, **f** Density plots of Spearman’s correlation coefficients of candidate metabolic drivers exhibiting significant correlation with the cancer-specific (**e**) and *global* list of previously reported cancer drivers (**f**). *BLCA* bladder urothelial carcinoma, *BRCA* breast invasive carcinoma, *COADREAD* colorectal adenocarcinoma, *GBM* glioblastoma multiforme, *HNSC* head and neck squamous cell carcinoma, *KIRC* kidney renal clear cell carcinoma, *LUAD* lung adenocarcinoma, *LUSC* lung squamous cell carcinoma, *OV* Ovarian serous cystadenocarcinoma, *UCEC* uterine corpus endometrial carcinoma

We next asked whether previously reported validated/putative cancer drivers could also account for metabolic dysregulation of the 44 core metabolic drivers or could be linked with the selection of SCNAs. To answer this, we compiled a comprehensive list of previously reported cancer drivers from three established sources [17–19] and tested whether our core metabolism signature was associated with the somatic mutational events underlying these drivers. Significant overlap (71 genes, *P* < 10^−4^; permutation analysis) was observed amongst the three sources of cancer drivers and a smaller but still significant overlap of four genes (*B4GALT3*, *CYC1*, *GMPS* and *SLC25A10*) was observed between the combined list from the three databases and the metabolic signature (*P* = 0.045, hypergeometric test) (Fig. 2a).

We further tested the likelihood of observing these previously reported cancer drivers (somatic mutations driven) in any of the 2752 metabolism genes and observed that metabolism genes were significantly enriched in the non-mutated component of the genome (the overlap with CGC drivers was 26 genes, *P* < 1 × 10^−5^; with Kandoth drivers it was seven genes, *P* = 1.4 × 10^−4^; with Tamborero drivers it was six genes, *P* = 4 × 10^−5^; 10^5^permutations; see the “Permutation analysis” section in the “Methods”). Since the cancer drivers in these three databases [17–19] were catalogued mainly based on somatic point mutations and indels (with the exception of CGC, which also contained amplifications, translocations and germline mutations), we assessed whether core metabolic signature genes exhibit SCNAs preferentially. By comparing the proportions of samples that had only SCNAs or only mutations, we conclude that core metabolic signature genes indeed have a tendency to harbour SCNAs as opposed to somatic mutations (Additional file 1: Figure S5d). This finding agrees with the previously illustrated *cancer genome hyperbola* model which depicts, on a whole genomic scale, SCNAs to be inversely correlated with mutational events [34].

Owing to this limited overlap between the metabolic signature and previously reported cancer drivers, we asked whether any association exists between the mRNA profiles of these cancer drivers and the metabolic signature (“Methods”). First, the background distribution of mRNA–mRNA correlation between random gene pairs was established for each cancer type (one million permutations; see the “Permutation analysis” section in the “Methods”; Fig. 2b; Additional file 1; Additional file 2: Table S10). Next, correlation analysis was performed between mRNA profiles of the core metabolic signature and mRNA profiles of (1) cancer-type specific candidate drivers [18] mutated in at least 5 % of the cohort and (2) a merged *global* high-confidence cancer gene consensus list [18, 19] (Additional file 1 and Additional file 2: Table S11). Cancer-type specific analysis revealed modest to strong correlation between the expression of previously reported cancer drivers and at least some of the 44 core metabolic drivers, except for OV, where no significant correlation was identified (Fig. 2c). Conversely, the analysis of the *global* list of 180 previously reported cancer drivers showed a correlation between a large proportion of these 44 genes and at least the expression of one cancer driver gene (Fig. 2d) and revealed a subgroup of these genes (Fig. 2d, orange covariate) significantly associated with expression of several previously reported cancer drivers across cancers (*P* < 0.05; 10,000 random permutations; see the “Permutation analysis” section in the “Methods”). These genes were enriched in drug, pyrimidine and purine metabolism (q values 8.34 × 10^−8^ to 0.0001, minimum overlap of three genes). In contrast, a second subgroup showed a weak correlation with expression of previously reported cancer drivers (Fig. 2d, blue covariate) and no enrichment in any biological pathway. In both analyses, inspection of the correlation values revealed a strong overall trend of inverse correlation between the expression of the 44 core metabolic signature genes and that of previously reported cancer drivers (Fig. 2e, f).

Finally, we compared the mRNA profiles of metabolic genes by stratifying them into mutant and wild-type groups using each of the cancer type-specific drivers. Significant differences (|log_2_ fold change| > 1, q value < 0.1) in mRNA abundance for eight metabolic genes (with sparse overlap between five cancer types) were observed, demonstrating limited dependence on the mutational status of previously reported cancer drivers (*TP53*, *ATRX*, *IDH1*, *NFE2L2*, *BAP1*, *USP9X* and *KEAP1*) (Additional file 1 and Additional file 2: Table S12).

In summary, these results indicate that SCNAs are the main events underlying over-expression of the 44 core candidate metabolic drivers, although they also suggest that previously reported (non-metabolic) cancer drivers may contribute to a dysregulated metabolism via indirect regulation of metabolic genes.

### Clinical relevance of the newly identified candidate metabolic drivers

**Fig. 3:**
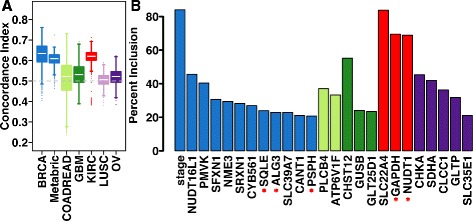
Prognostic significance of candidate metabolic drivers. **a** Distribution of concordance indices in held-out validation set/s using 1000 prognostic classifiers for each cancer type (see the “Survival analysis” section in the “Methods” ). The *dashed grey line* (concordance index = 0.5) represents a model with no prognostic ability. For breast cancer, models were trained using half of the TCGA cohort and tested on the other half as well as independently tested on the Metabric cohort. **b** Percentage inclusion of the most frequently chosen genes in each cancer type (>20 % of models) following univariate and LASSO feature selection performed on the training cohort. *Red asterisks* indicate genes present in the core metabolic signature

We asked whether the mRNA abundance profile of the 44 candidate metabolic drivers is a prognostic indicator, further substantiating gain of function. Univariate survival analysis of cancer type-specific candidate metabolic drivers revealed a number of genes that were prognostic (11 BRCA, two COADREAD, one GBM, three KIRC, two lung squamous cell carcinoma (LUSC) and six OV; *P* < 0.05, log-rank test; Additional file 1 and Additional file 2: Table S13). By pre-selecting prognostic genes (*P* < 0.1, log-rank test) over 1000 cross-validation iterations, we trained multivariate prognostic models including clinical variables for breast cancer and tested them on held-out subsets of the respective cohorts (see the “Survival analysis” section in the “Methods”). The BRCA (median concordance index = 0.63) and KIRC (median concordance index = 0.62) classifiers were consistently prognostic (1000 classifiers on randomly generated training sets) (Fig. 3a).

Importantly, BRCA prognostic classifiers were validated on an independent Metabric breast cancer cohort [15] (median concordance index = 0.61), indicating the robustness of the breast cancer classifiers. The BRCA classifiers were most frequently composed of clinical stage and three core metabolic signature genes (*SQLE*, *ALG3* and *PSPH*) whilst KIRC classifiers predominantly constituted two core metabolism genes (*NUDT1* and *GAPDH*) (Fig. 3b). COADREAD, GBM, LUSC and OV prognostic classifiers did not validate on their respective validation sets (Fig. 3a). Using the most frequently selected metabolism genes in breast cancer, a multivariate molecular classifier was created using the entire TCGA BRCA cohort and was successfully validated in the Metabric cohort (hazard ratio (HR)_Q4 vs Q1_ = 2.49, *P* = 1.11 × 10^−16^; Additional file 1: Figure S6a). Since clinical stage demonstrated a strong tendency of selection in the permutation analysis, we adjusted the molecular classifier for clinical stage and it remained an independent predictor of patient outcome (HR_Q4 vs Q1_ = 1.89, *P* = 6.45 × 10^−7^; Additional file 1: Figure S6b).

### Subtype-specificity and hypoxia association of metabolic drivers

**Fig. 4:**
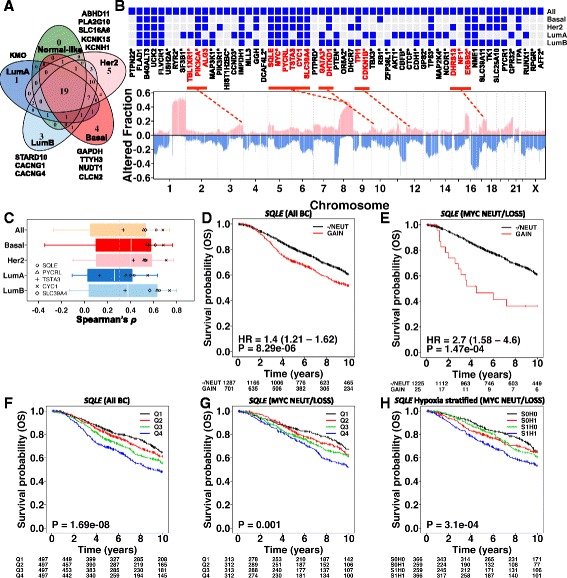
Independent validation of candidate metabolic drivers in the Metabric breast cancer cohort (*n* = 1991). **a** The overlap of candidate metabolic drivers identified in the TCGA BRCA cohort and tested for the correlation between mRNA and gene copy number data (log_2_ ratio) in the Metabric cohort. The *numbers* indicate correlated genes in the intrinsic subtypes of breast cancer (PAM50). Due to absence of subtype-specific candidate metabolic genes in the PAM50 subtype ‘*Normal-like’* breast cancer, it was not considered in subsequent analyses. **b** Genome-wide altered copy number fraction in the complete Metabric cohort (*n* = 1991). *Bottom*: *pink peaks* indicate fractional copy number gains/amplifications; *blue peaks* indicate fractional homozygous or heterozygous deletions. The *gene symbols* of candidate metabolic genes are located at the top in the order of their genomic location (*left* to *right*: chromosome 1 to X). *Gene symbols* with *asterisks* indicate significantly altered known breast cancer genes [25], *MYC* and *ERBB2. Genes in red* represent clusters of metabolic and significantly altered breast cancer genes that are in genomic proximity of each other. The *red dashed lines* show their approximate loci. *Top*: heat map showing the presence (*blue*) and absence (*grey*) of candidate metabolic genes across breast cancer intrinsic subtypes (PAM50) (as summarised in Fig. 4a). The subtype-specific significantly altered known breast cancer genes [25] are also highlighted with *asterisks*. **c** Correlation between mRNA and copy number data for genes in chromosome 8q24 amplicon using the complete Metabric cohort (*All*) and intrinsic subtypes of breast cancer. The candidate metabolic drivers are highlighted with *unique symbols* to show their mRNA dependence on gene dosage. **d** Copy number-based Kaplan–Meier analysis of *SQLE* in the Metabric breast cancer cohort. There were only five cases for which copy number state = heterozygous loss and, therefore, these were merged with the copy number diploid (*NEUT*, *n* = 1282) group. Genomic gains and amplifications were collapsed into one group (*GAIN*). **e** Same as (**d**) using the *MYC* diploid/loss subset of the Metabric breast cancer cohort. **f** mRNA-based Kaplan–Meier analysis of *SQLE* in the Metabric breast cancer cohort. Samples were split into four groups based on 75th percentile, median and 25th percentile of log_2_ mRNA abundance of *SQLE* (lowest = *Q1*, highest = *Q4*). **g** mRNA-based Kaplan–Meier analysis of *SQLE* in the *MYC* diploid/loss subset of the Metabric breast cancer cohort. **h** Median-dichotomised mRNA-based Kaplan–Meier analysis of *SQLE* further stratified into hypoxia high and low risk groups (*S0H0* = low *SQLE* and low hypoxia, *S0H1* = low *SQLE* and high hypoxia, *S1H0* = high *SQLE* and low hypoxia, *S1H1* = high *SQLE* and high hypoxia). *BC* breast cancer, *OS* overall survival

Cancer types and subtypes are heterogeneous [21, 24, 28]. We focused on breast cancer, where the presence of many different subtypes has been extensively reported [15], and also observed the strong association between hypoxia, genomic alterations and metabolism (Fig. 1; Additional file 1: Figure S1). We asked if genomic and clinical heterogeneity in breast cancer could be associated with metabolic differences. We used an independent cohort (Metabric) [15] further stratifying by well-known breast cancer subtypes (PAM50) [35]. Of the 103 (97 mapped to Metabric) candidate metabolic genes identified in the TCGA BRCA series (Additional file 1 and Additional file 2: Table S4), 49 maintained a positive correlation between mRNA abundance and copy number gains in the Metabric cohort (compared with 63 in the BRCA cohort) (*ρ* > 0.3, *q* < 10^−3^; Additional file 1 and Additional file 2: Table S14). The subtype-specific analysis revealed 19 core breast cancer metabolic genes present in all subtypes, as well as genes exclusive to aggressive subtypes (Basal-like, Her2-enriched and Luminal B; Fig. 4a; Additional file 1 and Additional file 2: Table S14). The number of genes showing positive mRNA–DNA correlations within each subtype was variable, with the highest in the aggressive subtypes: basal-like (50/97, *P* = 2 × 10^−4^) and Her2-enriched (52/97, *P* < 10^−4^) diseases (Additional file 1 and Additional file 2: Table S15).

The subtype-specific candidates showed some, although limited, overlap of five genes with previously reported cancer drivers (Additional file 1: Figure S7a–e). Next, we inspected the genomic distribution of breast cancer candidate metabolic drivers and previously reported cancer drivers. Examination of co-occurrence of genes in proximal chromosome bands revealed five clusters (which included nine metabolic genes) whereby genomic gains were significantly collocated. For instance, gain in chromosome 8q24 of *MYC* was significantly associated with *SQLE*, *PYCRL*, *TSTA3*, *CYC1* and *SLC39A4* (*P* = 0 to 3 × 10^−283^, Fishers exact test; Fig. 4b; Additional file 1 and Additional file 2: Table S16).

A similar trend was maintained in basal-like, Her2-enriched and luminal B breast cancers (*P*_*Basal*_ = 6.56 × 10^−53^ to 1.96 × 10^−38^, *P*_*Her2*_ = 5.48 × 10^−54^ to 4.37 × 10^−27^, *P*_*LumB*_ = 1.34 × 10^−106^ to 1.81 × 10^−78^; Fishers exact test; Fig. 4b; Additional file 1 and Additional file 2: Table S16), in line with previous studies demonstrating a link between *MYC* co-amplification and these genes as well as association with poor prognosis [36, 37]. Analysis of mRNA and DNA profiles on the chromosome 8q24 locus emphasised the importance of these five metabolism genes as they all demonstrated greater than average concordance between mRNA and DNA profiles (Fig. 4c).

As hypoxia is a driver of genomic gains (Additional file 1: Figure S1b, c) [32], we tested hypoxic association of SCNAs in these five genes, confirming significant positive correlation between SCNAs and the hypoxia signature [20] (Spearman’s *ρ*: *SQLE =* 0.31, *PYCRL =* 0.25, *TSTA3 =* 0.25, *CYC1 =* 0.25, *SLC39A4* = 0.24; *P* < 10^−3^).

Of the five *MYC* co-amplified candidate metabolic drivers, *CYC1* and *TSTA3* demonstrated significant association with poor survival using both DNA- and mRNA-based groupings (*CYC1*_SCNA_: HR = 1.26, 95 % confidence interval (CI) = 1.08–1.47, *P* = 0.003; *CYC1*_mRNA_: *P*_*quartiles*_ = 3.9 × 10^−5^; *TSTA3*_SCNA_: HR = 1.28, 95 % CI = 1.1–1.49, *P* = 0.002; *TSTA3*_mRNA_: *P*_*quartiles*_ = 0.037) but *SQLE* demonstrated the highest prognostic ability on both mRNA- and DNA-derived risk groups (Fig. 4d–g). Since we observed a modest increase in *SQLE* mRNA abundance in the *TP53* mutant group in breast cancer (Additional file 1 and Additional file 2: Table S12), we further interrogated this association with *TP53* mutation status in the TCGA breast cancer cohort (where all data modalities were available) and found that *SQLE’s* prognostic ability was decreased when adjusting for *TP53* mutation status (*P* = 0.03 to 0.18) (Additional file 1: Figure S8). However, *SQLE*’s mRNA association with prognosis was independent of *MYC* gain/amplification as we could recapitulate it in *MYC* neutral cases. Furthermore, following adjustment for clinical covariates (age, stage and grade), *SQLE* genomic classification remained an independent prognostic factor (HR = 3.2, 95 % CI = 1.64–6.26, *P* = 6.64 × 10^−4^) (Additional file 1 and Additional file 2: Table S17). Importantly, stratification of *SQLE*’s median-dichotomised (mRNA) risk groups by the hypoxia signature [20] indicated a worse prognosis group associated with *SQLE* over-expression in hypoxic tumours (Fig. 4h).

Next, we extended our breast cancer candidate metabolic drivers by repeating the analyses on each of the PAM50 subtypes in a large breast cancer series having sufficient representation of all subtypes (Additional file 1 and Additional file 2: Table S18). These data indicated 12 core genes common across all subtypes, including *SQLE* and *SLC39A4* (Additional file 1: Figure S7F) as core metabolic drivers of all breast cancers*.* We also tested these subtype-specific candidate metabolic genes for the presence of subtype-specific mutational events using the TCGA BRCA study [25]. Consistent with our previous observation of lack of mutational events in candidate metabolic drivers, none of the subtype-specific metabolic genes were significantly mutated.

In summary, our results indicate that the heterogeneity in breast cancer subtypes can be partially recapitulated by underlying metabolic signatures. These subtype-specific breast cancer metabolism signatures contained a common set of five genes (*SQLE*, *PYCRL*, *TSTA3*, *CYC1* and *SLC39A4*) which were co-amplified with *MYC* on chromosome 8q24 and showed a positive correlation with hypoxia. Of these genes, *SQLE* SCNA as well as mRNA profiles were associated with poor clinical outcome in an independent breast cancer cohort, with worse prognosis in hypoxic tumours.

### The top ranked, MYC co-amplified, metabolic driver *SQLE* is key for cell survival under hypoxic conditions

**Fig. 5:**
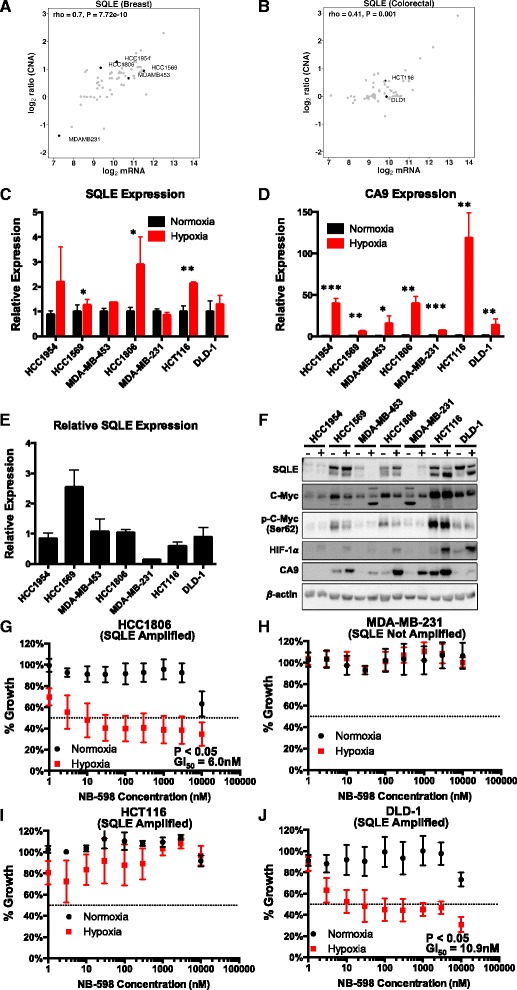
In vitro validation of *SQLE* is normoxia and hypoxia. **a** *SQLE* expression and copy number correlation in CCLE breast cancer lines. Cell lines used for in vitro validation are labelled. **b** *SQLE* expression and copy number correlation in CCLE colorectal cancer lines. Cell lines used for in vitro validation are labelled. **c** *SQLE* expression in cancer cell lines under 24-h normoxia and hypoxia (1 % O_2_). **d** *CA9* expression (hypoxia control) in cancer cell lines under 24-h normoxia and hypoxia (1 % O_2_). **e** *SQLE* expression in cancer cell lines relative to one another. **f** Western blot analysis showing the levels of *SQLE*, *MYC*, *phospho-MYC (Ser62)*, *HIF-1α* and *CA9* after 24 h of normoxia and hypoxia (1 % O_2_). The two *SQLE* bands correspond to different isoforms, which were further confirmed by siRNA knockdown western blots (Additional file 1: Figure S11q). **g–j** *SQLE* inhibition with NB-598 reduces cell viability at a range of concentrations with a GI50 of 6 nM in HCC1806 (**g**) and GI of 10.9 nM in DLD-1 (**j**) under hypoxia (1 % O_2_) but not normoxia. The effect of the compound was not inhibitory enough to calculate a GI50 value in MDA-MB-231 (**h**) or HCT116 (**i**) cells under normoxia or hypoxia (1 % O_2_). HCC1954 and HCC1569 are HER2+ breast cancer cell lines. MDA-MB-453, HCC1806 and MDA-MB-231 are triple receptor negative breast cancer cell lines. HCT116 and DLD-1 are colorectal cancer cell lines. Error bars are standard deviations. ***P < 0.001, **P < 0.01, *P < 0.05, *n* = 3

Focussing on the *MYC* locus (chromosome 8q24), we sought to elucidate the role of five candidate metabolism drivers (*SQLE*, *PYCRL*, *TSTA3*, *CYC1* and *SLC39A4*). All of these genes have shown varying but significant levels of essentiality in previous cell line shRNA screens [38] (Additional file 1 and Additional file 2: Table S19). Of these, *SQLE* was not only ranked amongst the top candidates in our analysis but also displayed putative gains/amplifications in breast cancer cell lines from the Cancer Cell Line Encyclopaedia (CCLE) [39] (Additional file 1: Figure S9a–d). We further verified *SQLE* copy number gains and mRNA correlation in both breast and colorectal cancer cell lines (Fig. 5a, b). In general, our findings from the clinical studies were corroborated in the CCLE by the correlation patterns between the DNA and mRNA profiles of candidate metabolism drivers (Additional file 1: Figure S10a–d). Given the positive correlation of five candidate metabolic genes located at *MYC* amplicon genes with the hypoxia gene expression signature in breast cancer (Spearman’s *ρ*: *SQLE =* 0.31, *PYCRL =* 0.25, *TSTA3 =* 0.25, *CYC1 =* 0.25, *SLC39A4* = 0.24; *P* < 10^−3^), we tested whether these five genes are directly induced by hypoxia and/or, when expressed, confer survival advantage under hypoxia. In vitro mRNA abundance analysis under hypoxia and normoxia revealed strong hypoxic dependence for *SQLE* in HCC1569 and HCC1806 breast cancer cells and HCT116 colorectal cancer cells (*P* < 0.05; unpaired *t*-test) (Fig. 5c, d). Analysis of *SQLE* protein expression confirmed hypoxic induction of *SQLE* in HCC1806 and, to a lesser extent, in HCC1569. None of the other chromosome 8q24 genes demonstrated hypoxic regulation (Additional file 1: Figure S11a–d). We validated the copy-number of *SQLE* and *MYC* in the breast and colorectal cell lines relative to the genome ploidy with quantitative PCR (Additional file 1: Figure S12). With the exception of HCC1954 and MDA-MB-231, all other cell lines had a significantly increased number of copies of *SQLE* (*P* < 0.05, unpaired *t*-test). The *MYC* copy number was also significantly increased (*P* < 0.05, unpaired *t*-test) in all cell lines except HCC1569 cells.

*SQLE* requires oxygen as a co-factor for its enzymatic reaction; thus, we hypothesised that it may show decreased activity in hypoxia and the selection of early genes in the cholesterol biosynthesis pathway for amplification in hypoxia, such as HMG-CoA reductase [40], may contribute to overcome the block. The increased amount of *SQLE* may compensate for decreased activity in hypoxia. The relative expression of *SQLE* at the mRNA (Fig. 5e) or protein (Fig. 5f) levels did not show any significant relationship to DNA copy number. This suggests other mechanisms such as epigenetic regulation, as shown previously [37], may also play a role in increasing *SQLE* expression in tumours.

**Fig. 6:**
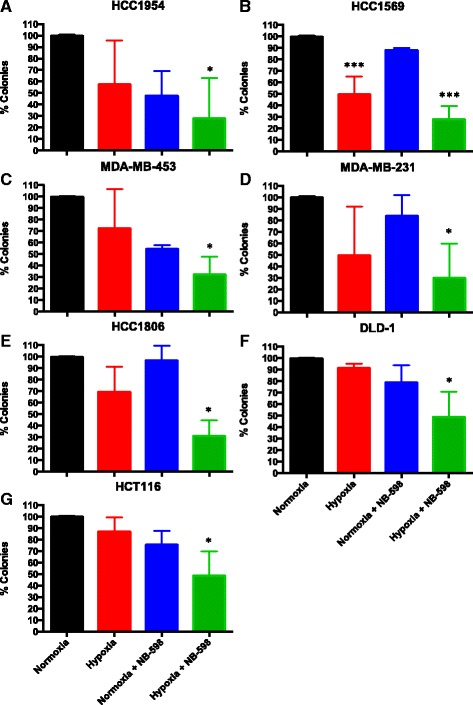
Clonogenic analysis of *SQLE.* **a–g** Clonogenic analysis of *SQLE* inhibition by NB-598 under normoxia and hypoxia (1 % O_2_) in HCC1954 (**a**), HCC1569 (**b**), MDA-MB-453 (**c**), MDA-MB-231 (**d**), HCC1806 (**e**), DLD-1 (**f**) and HCT116 (**g**) cells. ***P < 0.001, *P < 0.05, *n* = 3. *Black*, *red*, *blue* and *green bars* correspond to the percentage of colonies under normoxia and hypoxia and NB-598 cells under normoxia and hypoxia, respectively. Error bars are standard deviations

Given *SQLE*’s *MYC*-independent prognostic ability (Fig. 4e, g), association with hypoxia in breast and colorectal cancer samples (Spearman’s *ρ*: Breast-Metabric *=* 0.31, Breast-BRCA = 0.42, *P* < 10^−3^; COADREAD *=* 0.17, *P* = 0.003) and hypoxic induction in three cell lines amongst those tested (Fig. 5c), we focussed on functional analysis of *SQLE*. Breast and colorectal cancer cells were treated with SQLE inhibitor (NB-598) under hypoxia and normoxia (Fig. 5g–j). There was no effect on cell viability in GI50 assays under normoxic conditions, but the highly amplified triple negative breast cancer cell line HCC1806 and modestly amplified colorectal cancer cell line DLD-1 showed clear sensitivity to NB-598 under hypoxia (1 % O_2_; Fig. 5g, j). We further investigated the functional significance of *SQLE* by clonogenic assay (Fig. 6a–g). One cell line showed reduced survival in hypoxia alone (*P* < 0.001); however, in six of the seven cell lines tested, there was a significant synergistic effect of *SQLE* inhibition and hypoxia. Equivalent knockdown analyses on *TSTA3* and *PYCRL*, two of the other four top candidate metabolism genes, followed by cell viability and clonogenic assays indicated a smaller yet significant effect for *PYCRL* knockdown; in particular, *PYCRL* knockdown reduced the number of colonies in HCC1806 and DLD-1 cells only under hypoxia (*P* < 0.05; Additional file 1: Figure S11e–p).

In summary, *SQLE* expression in vitro demonstrated significant increases under hypoxia in three of the seven breast and colorectal cancer cell lines. *SQLE* was also confirmed as a driver of colorectal and triple receptor negative breast cancers and a key gene in maintaining cell survival under hypoxia.

## Discussion

The metabolism of rapidly dividing tumour cells is shifted towards increased anabolic processes and redox homeostasis compared with normal cells. Further, the hypoxic microenvironment adds additional metabolic stress, to which tumour cells have to adapt. Indeed, much research over the past decade has shown increased expression of and dependence on many genes involved in the tumour hypoxic microenvironment [41]. Moreover, complex interactions between oncogenic signalling pathways such as *MYC* and metabolism have been identified [2, 4, 6, 42]. Essential metabolic pathways hold potential to offer new druggable targets [43] and have served as therapeutic targets in a number of cancer types. In order to systematically identify new potential targets, we devised and exploited a novel in silico approach revealing metabolic dysregulation as an underlying source of selective genomic heterogeneity in a large pan-cancer study.

In a comprehensive analysis of 6538 samples from ten different cancer types, considering both genotypic (DNA) and phenotypic (mRNA and clinical prognosis) data , we show that over-expression of metabolic genes is substantially enriched with somatically acquired gains and amplifications. Our results suggest that these genomic alterations form a core part of the metabolic reprogramming that is conserved across different cancer types. Specifically, we identified a core signature of 44 metabolic drivers and revealed tumour site-specific metabolic genomic landscapes. We present evidence that differential metabolic requirements contribute to genomic heterogeneity amongst tumours and aggressiveness specifically in kidney and breast cancers. Core metabolic prognostic signatures are identified, comprising *NUDT1* and *GAPDH* in kidney renal cell carcinoma and *SQLE*, *ALG3* and *PSPH* in two independent breast carcinoma cohorts. Importantly, this identifies patients who might benefit from treatment with inhibitors of these genes, which in some cases are already being tested pre-clinically (e.g. *GAPDH* [44] and *NUDT1* [45] inhibitors).

Importantly, we show that both copy number amplification and expression of the identified metabolic driver genes are significantly associated with tumour hypoxia, suggesting a mechanism of metabolic reprogramming via adaptation within a hypoxic tumour microenvironment. We complement this analysis with evidence from in vitro and in vivo models supporting the hypothesis of selection pressure under hypoxia.

Amongst the top ranked candidates, we show *SQLE* is a metabolic driver in multiple cancers and demonstrate its association with prognosis and tumour hypoxia (Figs. 1 and 3). Importantly, high *SQLE* expression in hypoxic tumours was significantly associated with poor survival. *SQLE* is often co-amplified with the cancer driver *MYC* but *SQLE* expression is prognostic independent of *MYC* status. Furthermore, functional characterisation demonstrates *SQLE*’s specific role in maintaining proliferative integrity and regulating cell survival exclusively under hypoxia in a panel of cancer cell lines and independently of *MYC* amplification, confirming *SQLE* as a potential driver of selection under hypoxia. Interestingly, SQLE protein has also been detected in exosomes of breast cancer cells (MDA-MB-231) [46]. A recent study has also demonstrated that over-expression of *SQLE* promotes cell proliferation and migration and acts as a positive regulator of ERK signalling in hepatocellular carcinoma cells [47]. A second study, published while this manuscript was in revision, independently confirmed SQLE as bona fide oncogene by amplification, further supporting our findings [48]. *SQLE* catalyses a rate-limiting oxygenation step in sterol biosynthesis. Cholesterol is vital to cell membrane structure and also functions as a precursor of fat-soluble vitamins and steroid hormones [49]. It also forms part of lipid rafts which co-ordinate receptor-mediated signal transduction pathways [50]. Of the pan-cancer metabolic signature, *SQLE* and *DHCR7*, which catalyses cholesterol synthesis, were recently identified as the mediators of radiotherapy resistance in pancreatic cancer [51]. Hypoxic accumulation of squalene, the substrate of SQLE, occurs in glioblastoma, likely due to the dependence on oxygen as a co-factor for SQLE catalysis [52].

In summary, our study highlights a key role for hypoxia as a driver of genomic adaptation, including metabolic genes not directly induced by hypoxia and not previously identified as cancer drivers, such as *SQLE*. Importantly, drug inhibitors exist for several of the identified genes, allowing prompt translation and testing of these findings in clinical trials, with appropriate patient selection.

## Conclusions

Our pan-cancer analysis of genomic and transcriptomic profiles reveals a conserved landscape of metabolic dysregulation in human cancers linked with tumour hypoxia. We identified a core group of candidate metabolic driver genes as amplified and over-expressed across cancer types and strongly associated with tumour hypoxia in terms of both expression and DNA copy number. We also show that these metabolic drivers had not been previously identified as tumour suppressor and/or oncogenes and their over-expression in cancer cannot be explained by genomic alterations in known cancer drivers. Amongst the top candidate metabolic drivers for which drug inhibitors exist, we show that *SQLE* is independently prognostic and plays a key role in maintaining cell survival under hypoxia.

## Methods

### Pre-processing and filtering

mRNA abundance, DNA copy number and somatic mutation profiles for TCGA datasets were downloaded from TCGA DCC (gdac), release 2014-01-15. For mRNA abundance, Illumina HiSeq rnaseqv2 level 3 RSEM normalised profiles were used. Genes whereby >75 % of samples had zero reads were removed from the respective dataset. GISTIC v2 [53] level 4 data were used for copy number analysis and log_2_ ratios were processed to call SCNA states (human genome assembly Hg19). Briefly, SCNA gains/amplifications were based on 0: t < 0.2; 1: 0.2 ≤ t ≤ 1; 2: t > 1 and losses were based on 0: t > −0.2; −1: −0.2 ≥ t ≥ −1; −2: t < −1, where t denotes the log_2_ ratio. Level 1 clinical data were used for survival analysis. TCGA GBM and OV cancer microarray data were used for mRNA differential expression analysis as normal samples were not available on the Illumina HiSeq platform. mRNA data were converted to log_2_ scale for subsequent analyses. Analysis of differential mRNA abundance of candidate metabolic drivers stratified by mutational status of cancer type-specific drivers was based on TCGA DCC-reported MutSig v2.0 calls.

The Metabric breast cancer dataset was pre-processed, summarised and quantile-normalised from the raw expression files generated by Illumina BeadStudio (R packages: beadarray v2.4.2 and illuminaHuman v3.db_1.12.2). Raw Metabric files were downloaded from the European Genome-phenome Archive (EGA; study ID EGAS00000000083). Data files of one Metabric sample were not available at the time of our analysis and were therefore excluded. Probe to gene-level mapping was performed by keeping the most variable (standard deviation) probe. Log_2_ scaled data were used for differential expression analysis. Metabric copy number segmented genomic region data as published in the original study [15] (human genome assembly Hg18) were processed to create gene by patient log_2_ ratio and call profiles.

### Processing of xenograft samples

Mice were housed at BMS, University of Oxford, UK and procedures were carried out under a Home Office license. Six- to seven-week-old female BALB/c NuNu mice were injected subcutaneously in the lower flank with 100 μl Matrigel (BD Bioscience) and 1 × 10^7^ HCT116 cells suspended in 100 μl of serum-free medium. Tumour growth was monitored three times per week measuring the length (L), width (W) and height (H) of each tumour using calipers. Volumes were calculated from the formula 1/6 × π × L × W × H. For bevacizumab treatment mice were injected intraperitoneally every 3 days (10 mg/kg) until sacrifice once randomised groups had xenografts which had reached an average of 150 mm^3^ in size. When tumours reached 1.44 cm^3^ the mice were sacrificed by cervical dislocation.

### Pre-processing of Affymetrix CytoScan HD data

Affymetrix CytoScan HD array profiling was performed on three untreated HCT116-derived xenografts and three bevacizumab-treated samples. Data were normalised to a large collection of reference human normals as packaged with R package rawcopy (v1.0). Resulting logR ratio data were segmented using rawcopy’s default segmentation algorithm, PSCBS (Additional file 1 and Additional file 2: Table S20). Gene mapping to segmented regions was performed by rawcopy using RefSeq annotation. Since xenografts were exposed to bevacizumab for a limited period of time, we used relatively lenient logR ratio thresholds of ±0.1 for copy number gains and losses (Additional file 1 and Additional file 2: Table S21). Genes which were split in regions of gain as well as loss in the bevacizumab-treated groups were filtered out from subsequent analysis. Raw Affymetrix CEL files are available through the EBI Array Express portal (http://www.ebi.ac.uk/arrayexpress/) under accession number E-MTAB-4200.

### Fraction of transcriptome altered (genomic instability marker)

Genome instability in different gene sets (core metabolism signature, non-core metabolism, all metabolism and rest of the genome) was estimated at the gene level. For each gene, the fraction of patients with copy number calls 1,2 (gain/amplified) were regarded as the gained fraction and copy number calls −1,−2 were classed as the loss fraction. Across all gene sets, the fractions altered per gene were averaged to estimate an overall measure of the fraction of the cohort altered. This was estimated separately for gains and losses.

### Differential gene expression analysis

Differentially expressed genes were identified using Welch’s *t*-test. *P* values were adjusted for multiple comparisons using the Benjamini–Hochberg method. Genes with log_2_ fold change >0.5 and *q* < 0.05 (Benjamini–Hochberg method) were regarded as over-expressed, except for bladder urothelial carcinoma (BLCA), ovarian serous cystadenocarcinoma (OV) and uterine corpus endometrial carcinoma (UCEC), for which only a log_2_ fold-change >0.5 threshold was applied to keep the initial feature selection sets (top 10 % over-expressed metabolic genes) a similar size.

### Correlation analysis

All correlation analyses (mRNA–mRNA and mRNA–log2 copy number ratio) were performed using Spearman’s rank correlation.

### Pathway analysis

Pathway enrichment analysis for human samples was conducted using GeneCodis [54] against the KEGG database.

### Permutation analysis

The null distribution to estimate the enrichment of highly correlated metabolic genes at the mRNA and DNA levels was estimated by randomly selecting 10,000 genes from the non-metabolic section of the transcriptome. The gene set size was matched to the cancer type-specific size of genes exhibiting over-expression and gains/amplification (>20 %) in the metabolic genome.

Null distributions for mRNA–mRNA correlation for each cancer type were established by randomly sampling two distinct sets of 1000 genes, resulting in one million random correlations matched across cancer types. The reference distribution for mRNA–SCNA log2 profiles was established on common genes across all ten cancer types (14,923 genes).

Cancer-specific lists of candidate drivers were extracted from Kandoth et al*.* [18], further limiting the list to genes which showed >5 % mutation frequency in the respective cohorts. A *global* list of previously reported cancer drivers was compiled as a merged list of all Kandoth et al*.* genes and Tamborero et al*.* [19] putative drivers annotated as ‘High Confidence Driver’ and also present in the Cancer Gene Consensus [17]. For each cancer type, mRNA–mRNA correlations were estimated between core candidate metabolic drivers and the above-mentioned known candidate driver lists. Using the 95th and 5th percentile thresholds of null distributions for mRNA–mRNA correlations, correlation counts tables (Fig. 2c, d) were established. The probability values for each cell (Fig. 2c, d) were estimated by randomly sampling 10,000 subsets of genes and counting the number of times core candidate metabolic drivers showed correlation counts greater than observed. The same procedure was repeated for both cancer-specific candidate drivers (Fig. 2c) and the *global* list of candidate drivers (Fig. 2d).

Null distributions for overlap between metabolism genes and CGC, Kandoth et al*.* and Tamborero et al*.* drivers (as described previously) were estimated by generating 100,000 random subsets of genes from the non-metabolism part of the genome. Probabilities of observing an overlap with metabolism genes as low as 26 (CGC), 7 (Kandoth) and 6 (Tamborero) were subsequently estimated using the randomly generated counts.

### Co-occurrence analysis

We established a 2 × 2 counts table for each pair of genes using copy number call data. For each pairwise comparison, the table contained counts for when both genes had amplification, counts exclusive to one gene and counts for when neither had amplification. Due to different copy number-calling algorithms, for the Metabric dataset, gains and amplifications were treated as one category (amplifications). Fisher’s exact test was used to estimate the co-occurrence of copy number amplifications between a given pair of genes.

### Gene essentiality estimation

Gene essentiality estimates were generated in a panel of breast cancer cell lines using the COLT cancer database [38] (http://dpsc.ccbr.utoronto.ca/cancer). Data were filtered using default parameters, limited to breast cancer cell lines and a GARP (Gene Activity Ranking Profile) score *P* value <0.1.

### Cancer cell line encyclopaedia analysis

mRNA abundance and gene copy number log_2_ ratio data from the CCLE were downloaded from the Broad Institute (http://www.broadinstitute.org/ccle/). For gene copy number status, a threshold of 0.2 and –0.2 (consistent with thresholds used for patient data) was applied to log_2_ ratio profiles to determine putative gains/amplifications and losses, respectively. Breast cancer cell line subtype classifications were inferred from previously published data [55].

### Survival analysis

Survival modelling was limited to cancers with available survival data: BRCA, COADREAD, GBM, KIRC, LUSC, OV and Metabric. A Cox proportional hazards model was used to estimate the hazard ratio and a Wald test was used to test the significance of outcome difference between the risk groups. For univariate analysis, mRNA abundance profiles were median-dichotomised to established high-risk (high-abundance) and low-risk (low-abundance) groups. Overall survival time was truncated at 10 years for all cancers except for aggressive tumours (GBM, Metabric’s basal-like and Her2-enriched subtypes), where the truncation was done at 5 years.

For multivariate analysis, genes with correlated mRNA and copy-number profiles were selected by cancer type (BRCA, COADREAD, GBM, KIRC, LUSC and OV; Additional file 1 and Additional file 2: Table S5). Cohorts were randomly split into two halves (training and validation cohorts), except GBM, which was split 2:1 due to the small number of samples (*n* = 154). For each cancer type, genes were pre-selected through a univariate Cox proportional hazards model followed by log-rank test (*P* < 0.1; training cohort only). Selected genes were taken forward for multivariate Cox modelling with LASSO feature selection (*L1* penalty) in a tenfold cross-validation setting. The model with the smallest mean cross-validation error was selected and tested on the held-out validation cohort. For BRCA, all models contained clinical covariates of age (dichotomised at 50 years) and stage (1 = baseline, 2, 3, 4). These were also tested on the fully independent Metabric breast cancer cohort. Predicted response scores and predicted risk were evaluated for survival association in validation set/s using the concordance index.

Survival modelling was performed in the R statistical environment (v3.0.1) using the R packages survival v2.37-4, glmnet v1.9-5, glmpath v0.95, survcomp v1.10.0 and randomForestSRC v1.4.

### Cell culture, GI50 and clonogenic analysis

Cells were maintained in a humidified incubator at 5 % CO_2_ and 37 °C. For hypoxic exposure, cells were grown at 1 % O_2_, 5 % CO_2_ and 37 °C in an INVIVO_2_ 400 (Baker Ruskinn, USA).

MDA-MB-231, MDA-MB-453 and HCT116 cells were maintained in DMEM whilst HCC1806, HCC1954, HCC1569 and DLD-1 were maintained in RPMI, both supplemented with 10 % fetal bovine serum. *SQLE* inhibitor, NB-598, was available from MedChemexpress (USA).

For GI50 analysis, cells were plated in 96-well plates (1000 cells/well) and, 24 h later, NB-598 was added at a range of concentrations from 1 nM to 10 μM. The 96-well plates were then placed under normoxia or hypoxia for 72 h for the colorectal cancer cell lines (HCT116 and DLD-1) or 120 h for the breast cancer cell lines (HCC1954, HCC1569, MDA-MB-453, HCC1806 and MDA-MB-231). Cell viability at the start of treatment or at the endpoint was assessed using CyQuant (C7026, Life Technologies, UK) according to the manufacturer’s instructions. GI50 was calculated using PRISM (GraphPad, USA).

For clonogenic analysis, cells were plated in 100-mm dishes at 1000 cells/dish and 8 h later, once cells were attached, NB-598 was added at a concentration of 30 nM. Treated and untreated plates were incubated in normoxia or hypoxia for 72 h. After 72 h, cell culture medium was replaced with fresh medium followed by incubation in normoxia for 7–14 days until colonies were clear. The medium was removed and replaced with 0.5 % methylene blue dissolved in 50 % ethanol. Plates were incubated for 1 h at room temperature before washing in tap water. Colonies were visualised and counted utilising the GelCount™ system (Oxford Optronix, UK).

### Quantitative PCR

mRNA was extracted with Trizol (Invitrogen, CA, USA) according to the manufacturer’s instructions. DNA was extracted using DNAzol (Invitrogen, CA, USA) according to thr manufacturer’s instruction. Quantitative PCR was carried out as described previously [56] utilising the Applied Biosystems 7900HT Real-Time PCR System. Expression of genes was normalised against the expression of the control genes *RPL11* and *β-Actin*. The primer sequences for mRNA analysis were: *CA9* forward, CTTGGAAGAAATCGCTGAGG; *CA9* reverse, TGGAAGTAGCGGCTGAAGTC; *SQLE* forward, AAACGGGAGGCCTCTAAATC; *SQLE* reverse, AGATGGCCTCGGACTCAAG; *RPL11* forward, CTTTGGCATCCGGAGAAAT; *RPL11* reverse, TCCAGATTTCTTCTGCCTTG; *β-Actin* forward, CCAACCGCGAGAAGATGA; *β-Actin* reverse, CCAGAGGCGTACAGGGATAC; *PYCRL* forward GACAGGAACCTATGTCACTTTCAA; *PYCRL* reverse, TGGCAAAGATGACGAGCA; *TSTA3* forward, GAAGATGAGGTCTCCATCAAGG; *TSTA3* reverse, CCCATCCGACTTGGTTGTAT; *CYC1* forward, CTTCGCGGGGTAGTGTTG; *CYC1* reverse TTCGACGACAAGGCCACT; *SLC39A4* forward, CCTCTTCCTGCTGCACAAC; *SLC39A4* reverse, CATCCTCGTACAGGGACAGC. As chromosome 8, on which *SQLE* and *MYC* are located, is highly aberrant in breast cancer, control primers were designed from alternative chromosome regions for DNA copy number analysis. We identified regions which generally represented the genome ploidy of patient/cell line DNA. These were 2q11.1 (*TEKT4*) and 14q11.2 (*POTEG*). The primer sequences for DNA analysis were: *SQLE* forward, GCGTGCGACGGTTACTCT; *SQLE* reverse, GCTTCCTCACCAGCATCC; *MYC* forward, CGGTTTTCGGGGCTTTAT; *MYC* reverse, GGCTCTTCCACCCTAGCC; *PYCRL* forward, GGCACCACCATCTATGGACT; *PYCRL* reverse, CCTACTTTCTGCTGAGCTCCTT; *TSTA3* forward, AGCTGGAAGACAGGATCAGG; *TSTA3* reverse, AGAGCGGATGGAATGCAG; *POTEG* forward, GCGATCTGCTGGCTACTACC; *POTEG* reverse, CCAAATGGCTTCTTCACAGAG; *TEKT4* forward, GCTGACCACACACAGTCCTC; *TEKT4* reverse, GGGCCCACGTCGTCTTT.

### Western blots

Cell lysates were separated on 10 % SDS-PAGE and transferred to a PVDF membrane. Primary antibodies were used at 1:1000 unless otherwise stated. Antibodies against *CA9* (gift from J. Pastorek, Institute of Virology, Slovak Republic), *HIF1* (BD Biosciences, USA), *β-Actin* (A3854, Sigma, UK), *SQLE* (12544-1-AP, Proteintech, USA), *MYC* (9402, Cell Signalling, USA), *TSTA3* (HPA023301, Sigma, UK) and *PYCRL* (H00065263-M01) were utilised. Appropriate secondary horseradish peroxidase-linked antibodies were used (Dako, UK). Immunoreactivity was detected with chemiluminescence (Amersham, UK) and visualised using Image Quant LS4000 mini (GE Healthcare, UK).

### Visualisation

All visualisations were prepared in the R statistical environment (v3.0.1).

## Additional files

Additional file 1: Figures S1 to S12 including figure legends and supplementary references. (DOCX 35712 kb) Additional file 2: Tables S1 to S21. (ZIP 10219 kb) Additional file 3: iDOS R package and associated R scripts used in this study. (ZIP (379183 kb)
