# Supplementary material for: Genomic alterations underlie a pan-cancer metabolic shift associated with tumour hypoxia
Source: Genome Biol. 2016 Jun 29;17:140. doi: 10.1186/s13059-016-0999-8 (PMC4926297; doi:10.1186/s13059-016-0999-8)
Supplement: Additional file 1: — Figures S1 to S12 including figure legends and supplementary references. (DOCX 35712 kb) [file 13059_2016_999_MOESM1_ESM.docx]

## Additional file 1

### Figure S1. Relationship between hypoxia and genomic instability

**(A)** For each tissue type, hypoxia score was estimated using mRNA-based hypoxia signature [[1](#_ENREF_1)]. Our choice of hypoxia signature was driven by a recent comprehensive study assessing the robustness of different hypoxia signatures[[2](#_ENREF_2)]. We choose the best performing signature in that study which was a signature previously developed by our group and has been validated in several independent studies (reviewed in [[3](#_ENREF_3)]). The hypoxia signature score was compared between tumour (T) and normal (N) samples of each tissue type using two-sided Wilcoxon test, and P values are displayed. Tissue types were sorted on the basis of largest to smallest median hypoxia score in tumour samples.

**(B)** Mean (+/- standard error) of fraction of low-hypoxia (red) and high-hypoxia (blue) samples altered (gain/amplified) in metabolic genes. For each cancer type, hypoxia score was estimated using mRNA-based hypoxia signature. Patients in the top quartile were classed as Hypoxia-High group, and the patients in the bottom quartile were classed as Hypoxia-Low group. Difference between the low- and high-hypoxia groups was statistically quantified using one-sided Wilcoxon test; *p<0.05, **p<0.01, ***p<0.001. Test statistic is displayed only for those cancer types whereby high-hypoxia group showed higher mean genomic instability compared to low-hypoxia group.

**(C)** Same as **(B),** demonstrating genomic losses in metabolic genes.

**(D)** Mean (+/- standard error) of fraction of low-hypoxia (red) and high-hypoxia (blue) samples altered (Mutated) in metabolic genes. Statistical tests same as in **(B)**.

### Figure S2. Genome-wide copy-number profiles of xenografts with- and without Bevacizumab treatment

**(A)** Protein staining of two hypoxia markers in HCT116-derived xenografts (n=5 in each group). Cells were grown as mice xenografts and treated with Bevacizumab; an anti-angiogenic drug inducing hypoxia[[4](#_ENREF_4)]. Statistical comparison was performed using unpaired Wilcoxon test, confirming hypoxic induction in Bevacizumab-treated xenografts.

**(B)** Copy number aberrations as log R ratio of individual probes (in black) across the human genome. Of the ten samples in (A), six samples were selected for copy-number profiling and are displayed here (3 HCT116-derived xenografts, 3 HCT116-derived xenografts treated with Bevacizumab). Green lines show segmented mean log R ratio. Red box indicates chromosome 12 gains which were exclusive to Bevacizumab-treated xenografts.

**(C)** Fraction of altered genome in Bevacizumab-treated (Bev+) and untreated groups (Bev-) from HCT116-derived xenografts (n = 3) (Methods: Pre-processing of Affymetrix Cytoscan HD data). Data is displayed as total fraction altered as well as fraction altered due to copy-number gains and losses separately. These data demonstrated small but insignificant increase in the overall fraction of copy-number aberrations in Bevacizumab-treated samples (mean = 0.15, SD = 0.02, n=3) compared to untreated samples (mean = 0.12, SD = 0.004, n=3) (P=0.2, unpaired t-test) (Additional files 1 and 2: Table S20, segmented profiles). The increased aberrations were primarily contributed by the fraction of genomic gains (untreated mean = 0.056, SD = 0.002; Bevacizumab-treated mean = 0.082, SD = 0.02) (P=0.2, unpaired t-test) while the fraction of genomic losses remained unchanged (P=0.5, unpaired t-test). Genes with genomic gains exclusive to Bevacizumab-treated group (gain present in at least 2/3 Bevacizumab-treated samples and absent from all untreated samples) (Additional files 1 and 2: Table S21a), which represent early aberrations caused by tumour hypoxia.

**(D)** Overlap of genes between all metabolism genes and genes with copy-number gains induced in Bevacizumab-treated xenografts.

### Figure S3. Schematic overview of analytical and experimental strategy

**(A)** Stepwise overview of computational discovery process followed by prioritisation and experimental validation of pan-cancer metabolism signatures.

### Figure S4. Overview of pan-cancer metabolism signature

**(A)** Bar plot showing distribution of number of candidate metabolic genes identified in ten tumour types. Scatter plot showing enrichment analysis of candidate metabolic drivers. 10,000 random genesets were sampled matching the size of correlated genes (y-axis) observed in each cancer type. For each random geneset, correlation between mRNA and copy-number (log_2_ ratio) profiles was estimated, and probability values of expecting the observed correlation were computed (x-axis). Dashed gray line indicates P = 0.05.

**(B, C)** Pan-cancer mRNA abundance profiles and SCNA with rows representing genes and columns representing patients. mRNA profiles for each tumour type was *z*-transformed and patients were ordered using hierarchical clustering within each cancer separately. Somatic copy-number profiles of candidate metabolic drivers. Patients (columns) were ordered using hierarchical clustering.

### Figure S5. Hypoxic-dependence of candidate metabolic drivers

**(A)** Boxplot showing cancer-wise correlations (Spearman’s correlation coefficients) of copy-number (log2 ratio) profile of core metabolic signature (44 genes, ‘Sig.’ group) and mRNA-based hypoxia score, and compared to the correlations between non-selected metabolic genes (Other) and hypoxia score. For each cancer type, distribution of both sets of correlation coefficients was compared using one-sided Wilcoxon test with P values displayed. Box plots are sorted (high/left to low/right) by the median correlation coefficient in the signature (Sig.) group. To avoid bias, three genes common in metabolism signature and hypoxia signature, and another up to 17 genes (depending upon the cancer type) common in non-selected metabolic and hypoxia signature were removed.

**(B)** Mean (+/- standard error) of fraction of low-hypoxia (red) and high-hypoxia (blue) samples altered (gain/amplified) in core metabolic signature (44 genes). For each cancer type, hypoxia score was estimated using mRNA-based hypoxia profile. Patients in the top quartile were classed as Hypoxia-High group, and patients in the bottom quartile were classed as Hypoxia-Low group. For each cancer type, difference between the groups was statistically quantified using one-sided Wilcoxon test; *p<0.05, **p<0.01, ***p<0.001.

**(C)** Same as (B) except showing mean (+/- standard error) of fraction of low-hypoxia (red) and high-hypoxia (blue) samples altered through somatic mutations in core metabolic signature (44 genes). Note, COADREAD and UCEC had less than 3 samples in either low- or high-hypoxia groups due to small overlap of samples between mRNA and mutational profiles.

**(D)** Heatmap displaying difference in proportions of samples harbouring only SCNAs and only somatic mutations in the core metabolic signature. The significance of the difference was assessed using proportion test, and P-values (<0.05) are displayed as asterisks. Blank (empty) boxes correspond to the absence of that gene from the respective cancer type as shown in Figure 1A. Overall, all genes showed higher number of samples having just SCNAs and no mutations compared to mutation-only samples.

### Figure S6. Validation of breast cancer prognostic classifier

**(A)** Kaplan-Meier analysis of risk groups derived using the risk scores predicted by the multivariate molecular classifier based on 11 most frequently selected genes by TCGA BRCA cohort (see Figure 3B). Classifier was trained on TCGA BRCA cohort, and results of validation in Metabric are shown here. Risk groups (Q1-Q4) represents quartiles of predicted risk scores. Groups Q2-Q4 were compared to Q1 which was treated as a low-risk group. **(B)** Classifier’s performance was tested by adjusting the model for clinical stage.

### Figure S7. Drivers overlap between breast cancer subtype-specific candidate metabolism drivers

**(A)** Overlap between breast cancer candidate metabolism drivers (validated in Metabric cohort, discovered in TCGA cohort) and previously reported cancer drivers. The bold red title (All) indicates validation on complete Metabric cohort. The gene names in red italics indicate the overlapping genes between candidate metabolism genes (All) and each of the previously reported cancer drivers resource.

**(B-E)** Same as (A) but analyses were performed on each of the breast cancer subtypes (PAM50) separately.

**(F)** Overlap of candidate metabolic signatures discovered in PAM50 subtypes separately. Genes labelled in red indicate core breast cancer metabolism driver genes which were present in all subtypes.

### Figure S8. Survival analysis of *SQLE* in TCGA breast cancer cohort

**(A)** Copy-number-based Kaplan-Meier analysis of *SQLE* in TCGA breast cancer cohort. 18 cases with copy-number state = heterozygous loss were merged with copy-number diploid (NEUT, *n = 360*) group. Genomic gains and amplifications were collapsed in one group (GAIN).

**(B)** Same as (A) except the Cox model was adjusted for *TP53* mutation status (wild-type, mutant).

**(C)** Same as (A) except the Cox model was fitted to *TP53* wild-type cases only, to remove the effect of *TP53* mutation.

**(D)** mRNA-based Kaplan-Meier analysis of *SQLE* in TCGA breast cancer cohort. Samples were split into four groups based on 75^th^-percentile, median and 25^th^-percentile of log_2_ mRNA abundance of *SQLE* (lowest = Q1, highest = Q4).

**(E)** Same as (D) except the Cox model was adjusted for *TP53* mutation status.

**(F)** Same as (D) except the Cox model was fitted to *TP53* wild-type cases only, to remove the effect of *TP53* mutation.

### Figure S9. Breast cancer genomic gains/amplifications in cell lines

**(A,B,C,D)** DNA copy-number fraction of putative gains/amplifications (pink) and losses (blue) for all the genes in chromosome 8q24 in human breast cancer cell lines (CCLE). Each panel shows the fraction of cell lines altered in all breast cancer cell lines (A), basal-like (B), Her2-enriched (C) and Luminal B (D) cell lines respectively. Cell lines classification annotations[[5](#_ENREF_5)] did not map any cell line to Luminal A subtype. Candidate metabolism drivers and *MYC* are highlighted with red markers.

### Figure S10. DNA and mRNA level correlation of metabolism genes on *MYC* locus

**(A,B,C,D)** Spearman correlation between DNA (log2 ratio) and mRNA abundance data of candidate metabolism drivers on *MYC* locus, and *MYC* in human breast cancer cell lines (CCLE). Each panel shows the correlation plots in all breast cancer cell lines (A), basal-like (B), Her2-enriched (C) and Luminal B (D) cell lines respectively. Cell lines classification annotations[[5](#_ENREF_5)] did not map any cell line to Luminal A subtype. Red line shows the linear regression fit.

### Figure S11. *In-vitro* validation of metabolic genes on *MYC* locus

**(A-D)** The normoxic and hypoxic (1% O_2_) expression, at 24 hours, of **(A)** *TSTA3*, **(B)** *PYCRL*, **(C)** *SLC39A4* and **(D)** *CYC1* in a panel of cell lines.

**(E-H)** The relative expression of **(E)** *TSTA3*, **(F)** *PYCRL*, **(G)** *SLC39A4* and **(H)** *CYC1* for a panel of cell lines.

**(I)** The normoxic and hypoxic expression, at 24 hours, of *TSTA3* and *PYCRL*, - is normoxia + denotes 1% Oxygen in a panel of cell lines.

**(J,K)** The relative genomic copy-number of **(J)** *TSTA3* and **(K)** *PYCRL* in a panel of cell lines. Validation of *TSTA3* and *PYCRL* knockdown by siRNA in HCC1806 and DLD-1.

**(L-O)** The effect of *TSTA3* and *PYCRL* knockdown on cell viability over 72 hours in normoxia and hypoxia in **(L)** DLD-1 and **(M)** HCC1806. The effect of *TSTA3* and *PYCRL* knockdown in the clonogenic assay over 72 hours in normoxia and hypoxia in **(N)** DLD-1 and **(O)** HCC1806.

**(P)** *siRNA* knockdown western blots results of *TSTA3* and *PYCRL*. ***P<0.001, **P<0.01, *P<0.05, *n=3*.

**(Q)** *siRNA* knockdown western blots results of *SQLE* in hypoxia and normoxia. The two *SQLE* bands correspond to different isoforms of *SQLE* (UCSC Hg20 assembly). The top band shows the abundance of long isoform (574 amino acids) and bottom band shows the abundance of short isoform (479 amino acids).

### Figure S12. *MYC* and *SQLE* genomic copy-number relative to genome ploidy in a selected panel of cell lines

Statistical significance between normal DNA and each of the cell lines was estimated using t-test.

## References

1. Buffa FM, Harris AL, West CM, Miller CJ: **Large meta-analysis of multiple cancers reveals a common, compact and highly prognostic hypoxia metagene.** *Br J Cancer* 2010, **102:**428-435.

2. Fox NS, Starmans MH, Haider S, Lambin P, Boutros PC: **Ensemble analyses improve signatures of tumour hypoxia and reveal inter-platform differences.** *BMC Bioinformatics* 2014, **15:**170.

3. Harris BH, Barberis A, West CM, Buffa FM: **Gene Expression Signatures as Biomarkers of Tumour Hypoxia.** *Clin Oncol (R Coll Radiol)* 2015, **27:**547-560.

4. Rapisarda A, Hollingshead M, Uranchimeg B, Bonomi CA, Borgel SD, Carter JP, Gehrs B, Raffeld M, Kinders RJ, Parchment R, et al: **Increased antitumor activity of bevacizumab in combination with hypoxia inducible factor-1 inhibition.** *Mol Cancer Ther* 2009, **8:**1867-1877.

5. Prat A, Karginova O, Parker JS, Fan C, He X, Bixby L, Harrell JC, Roman E, Adamo B, Troester M, Perou CM: **Characterization of cell lines derived from breast cancers and normal mammary tissues for the study of the intrinsic molecular subtypes.** *Breast Cancer Res Treat* 2013, **142:**237-255.
