# Supplementary material for: Genomic alterations underlie a pan-cancer metabolic shift associated with tumour hypoxia
Source: Genome Biol. 2016 Jun 29;17:140. doi: 10.1186/s13059-016-0999-8 (PMC4926297; doi:10.1186/s13059-016-0999-8)
Supplement: Additional file 2: — Tables S1 to S21. (ZIP 10219 kb) [file 13059_2016_999_MOESM2_ESM.zip › Additional file 2 Captions.doc]

### Table S1. List of candidate metabolic genes

List of metabolic genes as previously published .

### Table S2. Overview of molecular datasets

List of discovery and independent validation (Metabric) datasets used for analyses. Numbers in parenthesis correspond to the microarray samples and genes used for differential expression analysis in GBM and OV. Column ‘Top 10% Over-Expressed Genes, with Gain Fraction > 20%’ shows the number of top 10% over-expressed metabolic genes, which also had gain/amplification fraction > 20%. Genes with RNA-seq RSEM normalized read count ≤ 1 in > 75% of tumour samples, were discarded. Column ‘Number of events’ shows the overall survival associated events in the respective cohort. Metabric breast cancer dataset was strictly used as validation cohort of candidates identified using TCGA BRCA cohort, and six candidate gene symbols did not map between BRCA (103 genes) and Metabric (97) datasets.

### Table S3. Differentially expressed genes

Cross-cancer differential gene expression analysis with log_2_ fold change and adjusted P-values (see Methods). NA entries correspond to the genes which were either not present on the platform (microarray) OR dropped due to lack of abundance in RNA-seq data.

### Table S4. Spearman’s correlation statistics of candidate metabolic genes

Cross-cancer Spearman’s correlation statistics of genes (Table S1) that passed the pre-selection criteria of over-expression and gained fraction, as listed in Table S2.

### Table S5. Pan-cancer inclusion frequency of candidate metabolic genes

Cross-cancer inclusion frequency of genes whereby mRNA and copy-number profiles are significantly correlated (*ρ* > 0.3, *q* < 10^-3^). ‘1’ represents presence of correlated genes, while ‘0’ represents genes failing the above-mentioned criteria. Highlighted in green are the genes present in at least 3 cancer types.

### Table S6. Core metabolism signature

Gene symbols and description of core metabolic candidate driver genes which were present in at least three cancer types.

### Table S7. Enrichment analysis

Enrichment analysis of core metabolic candidate drivers against KEGG database using GeneCodis.

### Table S8. Pan-cancer mutation frequency of core signature

**(a)** Pan-cancer mutation frequency data of core metabolic candidate drivers expressed as percentage of respective cohorts. BLCA was not including in the estimation of ‘Pan-cancer Mean %’ due to small sample size (n=28). (b) Same as (a) but for all metabolic genes.

### Table S9. Metabolic signature derived using genes down-regulated and having genomic loss/deletion

Loss of function genes that exhibited down-regulation in tumour samples compared to normal samples, loss in 20% of respective cohort and having mRNA and copy-number profiles correlation (*ρ* > 0.3, *q* < 10^-3^). ‘1’ represents presence of correlated genes, while ‘0’ represents genes failing the above-mentioned criteria. Highlighted in green are the genes present in at least 3 cancer types.

### Table S10. Genome-wide SCNA-mRNA correlations

Summary of Spearman’s correlation coefficients of genome-wide mRNA and copy-number (log_2_ ratio) data for all cancer types. Percentages in first column represent the respective percentiles values.

### Table S11. mRNA-mRNA correlations between candidate metabolism drivers and previously reported cancer drivers

Spearman’s correlation coefficients and P-values of mRNA-mRNA correlation between core metabolic driver genes (rows) and 1) cancer-type specific candidate drivers (a: correlation coefficient, b: P value) and 2) global list of previously reported cancer drivers (c: correlation coefficient, d: P value). Columns contain cancer-type specific and global drivers.

### Table S12. Differential mRNA abundance analysis of candidate metabolic drivers stratified by mutational status

Cross-cancer differential mRNA expression analysis of core metabolic drivers between mutant (MT) and Wild-type groups established based on cancer-type specific mutational drivers (see Table S11a for the list of drivers). For each cancer type, column ‘Mutant vs Wild-type Driver’ represents the name of the metabolism genes (whose expression is compared) and the name of the driver gene used to derive Mutant and Wild-type groups. log_2_ fold change (Mutant – Wild-type) and adjusted P-values (two-sided Wilcoxon test; Benjamini & Hochberg method) are reported as additional columns. Highlighted in green are those comparison whereby |log_2_FC| > 1 and adjusted P < 0.1. Analysis was limited to samples with matched mRNA expression (RNA-seq data) and mutational profiles. Cancer drivers resulting in < 3 samples in either Mutant or Wild-type groups were removed from the analysis (this was the case for all of the COADREAD and UCEC drivers).

### Table S13. Univariate survival analysis

Univariate survival analysis of all the genes exhibiting correlation between mRNA and copy-number (log_2_ ratio) data in a given cancer type. Cox model was fitted on median-dichotomised mRNA abundance profiles (see Methods). Genes highlighted in green are the most frequently selected genes by the multivariate prognostic models as shown in Figure 3B.

### Table S14. Independent validation in breast cancer subtypes

Independent validation of TCGA BRCA derived metabolic genes in Metabric breast cancer cohort. Out of 103 BRCA genes, 97 successfully mapped to Metabric dataset. Spearman correlation coefficients were estimated between mRNA and copy-number (log_2_ ratio) data. P-values were adjusted for multiple comparisons (column [SubtypeName].Q). ‘All’ represents data using complete Metabric cohort. Same analysis was repeated on intrinsic subtypes of breast cancer (PAM50).

### Table S15. Enrichment of metabolic signature in breast cancer subtypes

Enrichment analysis of genes exhibiting correlated mRNA and copy-number profiles in complete Metabric cohort and on intrinsic subtypes (PAM50) of breast cancer. 10,000 random subsets of 97 genes were generated, and probability of expecting the observed correlations (column: Observed) in each subtype was estimated (column: P).

### Table S16. Co-occurrence of SCNAs between candidate metabolism drivers and previously reported cancer drivers

Co-occurrence of copy-number events (gains/amplifications) between a pair of metabolic candidate drivers and significantly mutated candidate drivers of breast cancer, *MYC* and *ERBB2*. P-values from Fishers exact test on 2x2 counts table are reported (see Methods). Analysis was performed on complete Metabric cohort (a) as well as Basal-like (b), Her2-enriched (c), Luminal A (d), Luminal B (e) subtypes using their respective list of significantly mutated candidate drivers list.

### Table S17. Multivariate survival analysis

Multivariate Cox proportional hazards model to assess prognostic value of *SQLE* mRNA abundance and *SQLE* copy-number states. Models were adjusted for age (dichotomised at 50yrs), stage (1=baseline, 2, 3, 4) and grade (1 = baseline, 2, 3).

### Table S18. Breast cancer subtype-specific candidate metabolic signatures

Spearman’s correlation statistics of genes that passed the pre-selection criteria of over-expression and gained fraction consistent with cross-cancer discovery procedure (Table S2). Discovery was performed using each of the PAM50 subtypes separately (Metabric cohort).

### Table S19. Gene essentiality analysis

Gene essentiality estimates reflecting relative importance of candidate metabolism genes and *MYC* in a range of breast cancer cell lines. Lower GARP score indicates higher essentiality and vice versa.

### Table S20. Segmented data of HCT116 derived xenografts.

**(a-c)** HCT116 derived xenograft samples without treatment. **(d-f)** Bevacizumab treated xenografts.

### Table S21. Copy number calls of HCT116 derived xenografts

**(a)** Copy number calls for genes in regions with genomic gains. Presence of gains is shown by ‘1’ in control (Bev-) and Bevacizumab treated (Bev+) xenografts. Recurrent gains are shown as number of samples exhibiting that genomic gain in ‘Sum Bev-/Bev+’ columns. **(b)** Same as (a) but showing copy number losses.

## References

1.	Possemato R, Marks KM, Shaul YD, Pacold ME, Kim D, Birsoy K, Sethumadhavan S, Woo HK, Jang HG, Jha AK, et al: **Functional genomics reveal that the serine synthesis pathway is essential in breast cancer.** *Nature* 2011, **476:**346-350.

2.	Tabas-Madrid D, Nogales-Cadenas R, Pascual-Montano A: **GeneCodis3: a non-redundant and modular enrichment analysis tool for functional genomics.** *Nucleic Acids Res* 2012, **40:**W478-483.

3.	Cancer Genome Atlas N: **Comprehensive molecular portraits of human breast tumours.** *Nature* 2012, **490:**61-70.
